# Long-term treatment with dehydroepiandrosterone may lead to follicular atresia through interaction with anti-Mullerian hormone

**DOI:** 10.1186/1757-2215-7-46

**Published:** 2014-04-30

**Authors:** Keiko Ikeda, Tsuyoshi Baba, Miyuki Morishita, Hiroyuki Honnma, Toshiaki Endo, Tamotsu Kiya, Tsuyoshi Saito

**Affiliations:** 1 Department of Obstetrics and Gynecology, Steel Memorial Muroran Hospital, 1-45, Chiribethutown, Muroran, Hokkaido 050 0076, Japan; 2 Department of Obstetrics and Gynecology, Sapporo Medical University, South 1 West 16, Sapporo, Hokkaido 060 8543, Japan; 3 Kamiya Ladies Clinic Sapporo, North 3 West 2, Sapporo, Hokkaido 060 0003, Japan; 4 Ena Ladies Clinic, 9-1-86 Hanakawa-minami, Ishikari, Hokkaido 061-3209, Japan

**Keywords:** Polycystic ovary, Animal model, Anti-mullerian hormone, Folliculogenesis, Androgen

## Abstract

**Background:**

Hyperandrogenism is the primary manifestation of polycystic ovary syndrome (PCOS), which appears to be caused by excess exposure to androgen. As such, androgenized animal models have been developed and investigated to study the etiology of PCOS. Anti-Mullerian hormone (AMH) is known to be associated with follicle growth, and its levels are two to three times higher in women with PCOS than in those with normal ovaries. We studied how duration of androgen administration affects folliculogenesis and AMH expression.

**Methods:**

We divided 30 immature (3-week-old) Sprague Dawley rats into six groups. Three groups were injected each evening with dehydroepiandrosterone (DHEA) (6 mg/100 g body weight/0.2 ml sesame oil) for 7, 15 or 30 days, respectively. The three control groups were injected with 0.2 ml of sesame oil for the corresponding lengths of time. Resected ovaries were sectioned and examined to determine follicle numbers at each developmental stage, and immunostained to assess AMH expression.

**Results:**

On day 7, follicle numbers and AMH expression levels at each developmental stage of follicle growth were similar in the respective control and DHEA groups. On day 15, the total follicle number (P = 0.041), the percentage of primordial follicles (P = 0.039) and AMH expression were significantly greater in the DHEA than the control group. On day 30, the percentages of primordial (P = 0.005), primary (P = 0.0002) and atretic (P = 0.03) follicles were significantly greater in the DHEA group, whereas the percentage of intermediary follicles (early pre-antral, late preantral, and early antral follicles) was significantly lower in the DHEA group (P = <0.0001). AMH expression in DHEA-treated rats on day 30 was seen exclusively in the primordial (P = 0.0413) and late antral follicles (p = 0.028).

**Conclusions:**

Androgen administration increases AMH production in a process that regulates the growth of primordial follicles. That is, androgen-induced AMH expression provides local negative feedback to folliculogenesis augmented by androgen.

## Background

Polycystic ovary syndrome (PCOS) is the most common endocrine-metabolic dysfunction in women of reproductive age, and a frequent cause of anovulatory infertility and hyperandrogenism [1]. The polycystic ovary (PCO) morphology in PCOS is characterized by an excessive number of growing follicles, suggesting folliculogenesis is altered in this syndrome [2]. Hyperandrogenism is the primary manifestation of PCOS, and it is the excess androgen exposure that appears to be the cause of the PCO morphology. However, we have shown that long-term administration of androgens for female-to-male transsexual (FTM) persons leads to an increase in the number of atretic follicles, not to the PCO morphology [3].

Several androgenized animal models have been developed and investigated to determine the etiology of PCOS. Slight differences among the models reflect differences in the timing and duration of the androgen administration, as well as the amount of hormone administered [4-6]. Nearly all animals administered androgen during fetal life exhibit PCO and show endocrine abnormalities similar to PCOS [4,7]. In a report on the effects of androgen administration to immature rats, short-term treatment caused PCO morphology, while medium-term treatment resulted in an increase in follicle atresia [6]. In another study, reproductive-age rhesus monkeys receiving high doses of androgen in the short-term were equally as likely to exhibit an increase in small follicles as monkeys receiving low doses over a longer term [5]. In addition, examination of the histology of ovaries in androgen-administered FTM patients revealed increased follicular atresia, though the morphogenesis of polycystic ovaries is controversial [3,8,9]. Judging from these findings, it appears that androgen administration may impact folliculogenesis and subsequent ovarian morphology, leading to the development of the PCO morphology, but the effects of exogenous androgens on ovarian morphology may vary depending on the period and duration of the androgen administration and the amount of hormone administered. In other words, androgen-induced PCO morphology may be a time-lapse snapshot of metamorphosis.

Because the effect of androgen is not constant, other factors modifying its effects on folliculogenesis may exist. For example, we have been particularly interested in anti-Mullerian hormone (AMH), which is known to be associated with follicle growth. AMH is produced by granulosa cells in early developing follicles and inhibits the transition from the primordial to the primary follicular stage. AMH levels can be measured in serum and have been shown to be proportional to the number of small antral follicles [10]. In women with PCOS, serum AMH levels are two to three times higher than in women with normal ovaries [11]. This is thought to be due to the large number of small antral follicles as well as enhancement of the AMH production per granulosa cell [12]. On the other hand, Stubbs et al. reported that primordial follicles only weakly express AMH, and that expression levels of AMH in primordial follicles in PCOS are lower than in the control [13]. In addition, prenatally androgenized sheep, which manifest PCOS-like features, exhibited reduced AMH expression by preantral and antral follicles [14]. In PCOS, the relationship between hyperandrogenism and AMH remains unclear. It is thought, however, that androgen and AMH are closely related, and both affect folliculogenesis.

In the present study, we sought to determine the effect of the duration of androgen administration on folliculogenesis and AMH expression using a juvenile rat model. Our primary aim was to clarify the roles of androgen and AMH in the pathogenesis of PCOS.

## Methods

### Animals and treatments

The study was carried out using 30 immature (3-week-old) Sprague Dawley rats, with body weights of 35–40 g and regular (4–5 days) ovulatory cycles. The rats were injected (subcutaneously) with dehydroepiandrosterone (DHEA; Wako Hormone Manufactory Co., Tokyo, Japan) (6 mg/100 g body weight/0.2 ml sesame oil) each evening for 7,15 or 30 days (7-, 15- and 30-day DHEA groups, respectively). Control rats were injected with 0.2 ml of sesame oil each evening for the corresponding lengths of time (7-, 15- and 30-day control groups, respectively) [6,15]. Rats were sacrificed by decapitation on the evening of day 8, 16 or 31. Animals used in this study were maintained in accordance with the guidelines of the Animal Resources Centre of the Sapporo Medical University School of Medicine.

### Immunohistochemistry

For immunohistochemical analysis, ovaries were dissected, weighed, fixed in 10% formalin, embedded in paraffin, and serially sectioned longitudinally throughout the largest diameter of the ovaries (thickness, 7 μm). One slice from each ovary was stained with hematoxylin-eosin for a follicle count. Then after deparaffinization in xylene and rehydration through an ethanol series, the other sections were placed on slides, and AMH expression was detected immunohistochemically. Initially, antigens were retrieved using Histo VT one (10×, pH 7.0; Nacalai Tesque, Inc., Kyoto, Japan), and nonspecific antibody binding was reduced by incubation for 60 min with 1% normal rabbit serum in PBS. The primary antibody, goat anti-human AMH (Santa Cruz Biotechnology, Santa Cruz, CA, USA), was diluted 1:50 in PBS containing 1% normal rabbit serum and incubated with the sections overnight at 4°C. Thereafter, the sections were incubated for 30 min with biotinylated secondary antibody, followed by incubation with ABC reagent (Vectastain ABC Kit, Vector Laboratories, CA, USA) for 5 min at room temperature. Diaminobenzidine (DAB) was used as a chromogen (Peroxidase Stain DAB Kit, Nacalai Tesque, Inc., Kyoto, Japan). The sections were counterstained with Mayer’s hematoxylin.

### Morphological classification and analysis

A single investigator blinded to the weekly age and treatment of the rats assessed the follicle number and the presence or absence of staining. The numbers of follicles at each developmental stage in each group were estimated by counting follicles in each section per individual. The criteria used for classifying the developmental stages of follicles were as follows: 1) primordial follicle, oocyte surrounded by a single squamous layer of follicular somatic cells; 2) primary follicle, a single layer of cuboidal granulosa cells; 3) early preantral follicle, two to four layers of granulosa cells; 4) late preantral follicle, more than four layers of granulosa cells and no antrum; 5) early antral follicle, contains an antrum and the follicle diameter is < 220 μm; 6) late antral follicle, contains an antrum and the follicle diameter is > 220 μm; 7) atretic follicle, contains a degenerate oocyte, an oocyte that has resumed meiosis, or cumulus cells dissociated from the oocyte, and pyknotic granulosa cells are common [16].

**Figure 1:**
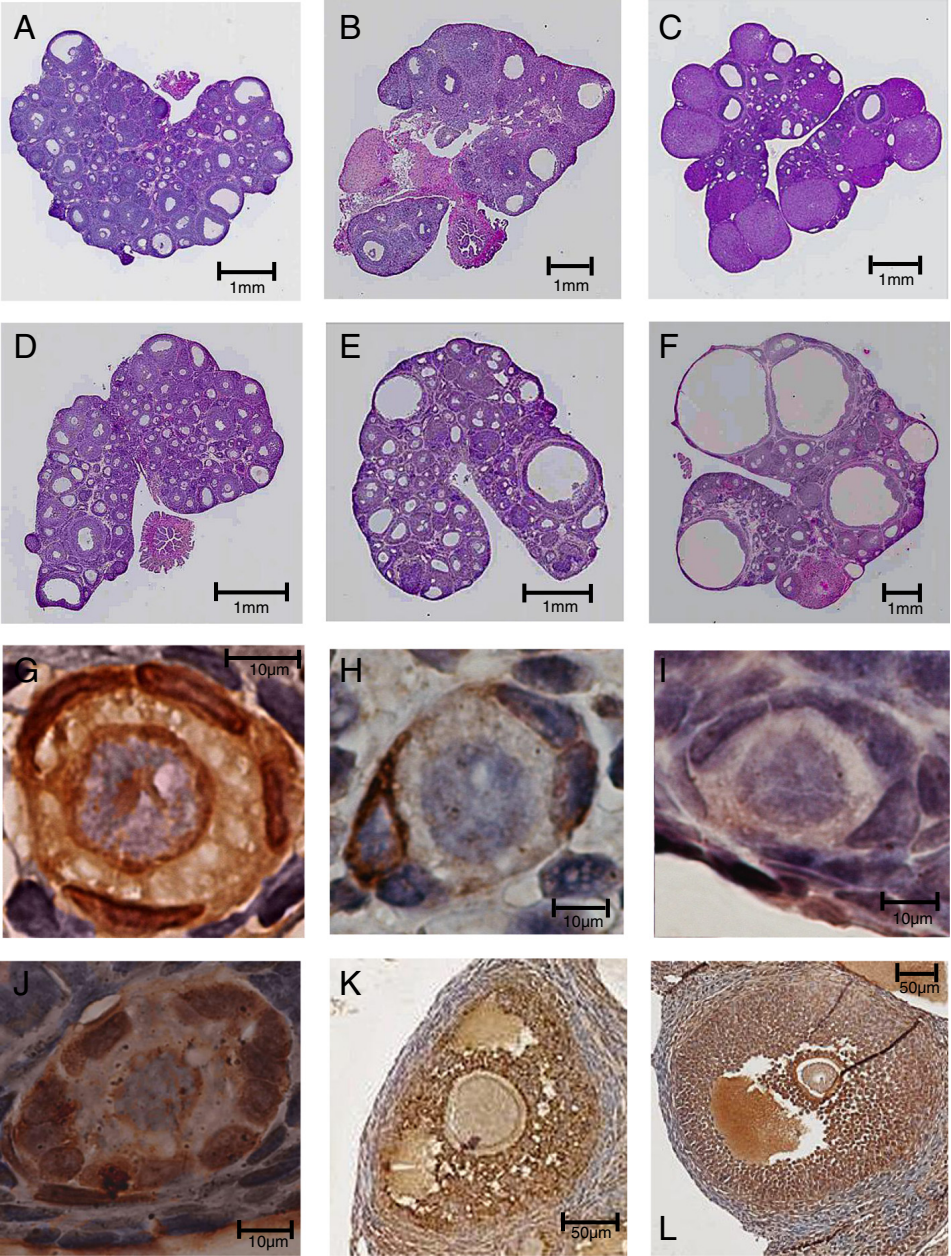
**Ovaries and follicles immunostained for AMH in DHEA-treated and control rats. (A ~ F)** Light micrographs of hematoxylin/eosin-stained whole rat ovary sections: vehicle (control) administration for 7 days **(A)**, 15 days **(B)** or 30 days **(C)**; DHEA administration for 7 days **(D)**, 15 days **(E)** or 30 days **(F)**. At 7 days, the ovaries of both groups **(A, D)** contain numerous follicles and were similar in configuration. The day-15 DHEA ovary has more small follicles than control ovaries **(B, E)** and exhibits a polycystic-like morphology. Day-30 control ovaries have fewer follicles than any of the other groups **(C)**. Day 0-30 DHEA ovaries have more cystic follicles than ovaries of the day-30 control and day-15 DHEA groups **(C, E, F)**. These micrographs are at different magnifications. **(G ~ L)** AMH immunostaining in ovaries from DHEA-treated and control rats: **(G)** Strong diffuse expression of AMH (+) in primordial follicles; **(H)** Mild sparse expression (±) in primordial follicles; **(I)** No expression (−) in primordial follicles; **(J)** Strong expression in primary the follicles; **(K)** Moderate expression in the late preantral follicles; **(L)** Strong expression in early antral follicles.

AMH staining was assessed only in healthy follicles. Atretic follicles with pyknotic granulosa cells or oocytes were excluded from immunohistochemical analysis. The intensity of AMH staining was determined on screen using a printed example of each follicle for relative comparison, and was classified according to the following criteria: −, no staining; ±, mild to moderate staining; and +, strong staining (Figure 1-G-I). To calculate the AMH staining score, − = 0, ± = 1, and + = 2. The calculated scores per 100 follicles in the DHEA and control groups were compared.

### Statistical analysis

Data were analyzed using Excel Statistics, 2009 (OMS publishing company, Tokorozawa, Japan). The quantitative results (follicle number and hormonal parameters) are presented as median [interquartile range (IQR)] and were compared using nonparametric analysis (Mann–Whitney U test). The proportions (the percentage of follicles at each stage and the percentage of follicles expressing AMH) and AMH expression scores were compared using the χ^2^ test.

## Results

### Histopathological analysis

**Figure 2:**
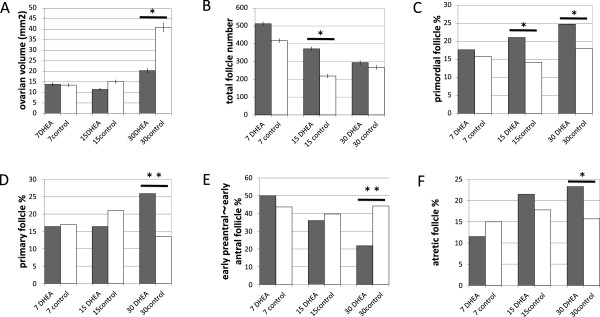
**Comparison of ovarian area, follicle numbers and follicles at each stage. (A)** Mean ovarian volume (mm^2^) in DHEA-treated vs. untreated ovaries. The height, major axis and minor axis of each ovary were measured in 0.01 mm increments; ovarian volumes were then calculated using the formula**,** Volume = (4 π a b c) / 3, where a = height, b = length of the major axis, and c = length of the minor axis. Values are medians ± 95% confidence intervals of the median. Average ovarian volumes were significantly smaller than control in the day-30 DHEA group. **(B)** Total number follicle numbers were significantly higher than control in the day-15 DHEA group. **(C)** Percentages of primordial follicles were significantly higher than control in the day-15 and day-30 DHEA groups. **(D)** Percentages of primary follicles were significantly higher than control in the day-30 DHEA group. **E)** Percentages of intermediary follicles (early preantral, late preantral and early antral) were significantly lower than control in the day-30 DHEA group. **(F)** Percentages of atretic follicles were significantly higher than control in the day-30 DHEA group. *P < 0.05; **P < 0.01.

The macroscopic ovarian findings on day 7 were similar in both the DHEA and control groups (Figure 1A, D). On day 15, by contrast, ovaries in the DHEA group exhibited PCO morphology (Figure 1E), but the control group did not (Figure 1B). Ovaries also showed PCO-like morphology in the day-30 DHEA group, but with larger cystic follicles than were seen on day 15 (Figure 1E, F). In the day-30 control group, ovaries had fewer follicles than in other groups (Figure 1C). On days 7 and 15 (juvenile period), ovary volumes and weights were similar in the DHEA and control groups. On day 30 (pubertal period), by contrast, ovary volumes and weights in the DHEA group were smaller than in the control group. Ovaries in the day-30 DHEA group showed no increase in weight, as compared to the day-15 DHEA group, despite an increase in overall body weight (Figure 2A).

Approximately 1773 follicles were examined in 30 sections from the ovaries of the 30 rats. In the day-7 groups, total follicle number and the percentages of follicles at each developmental stage were similar (Figure 2B-F). In the day 15 groups, total follicle number (P = 0.041, Figure 2B) and the percentage of primordial follicles (P = 0.039, Figure 2C) were significantly greater in the DHEA group than the control group. In the day 30 groups, total follicle numbers were similar in the DHEA and control groups (Figure 2B), but the percentages of primordial (P = 0.005, Figure 2C), primary (P = 0.0002, Figure 2D) and atretic (P = 0.03, Figure 2F) follicles were significantly larger in the DHEA group, while the percentage of intermediary follicles (early preantral, late preantral, and early antral follicles) was significantly smaller in the DHEA group than the control group (P < 0.0001, Figure 2E).

### Immunolocalization of AMH

AMH is expressed in granulosa cells. Although oocyte morphology was poorly preserved in these formalin-fixed sections, AMH immunostaining was detected in more than half of the oocytes. Low levels of AMH staining were detected in primordial follicles (Figure 1G, H), and a very small number of follicles showed strong staining (Figure 1G). Mild to moderate staining was observed in more than half of the primary follicles (Figure 1J), while moderate to strong staining was observed in a majority of intermediary follicles (early preantral, late preantral, and early antral) (Figure 1K, L), though expression was diminished in late antral follicles. Atretic follicles exhibited irregular staining of the granulosa cells still adhering to the basal lamina (data not shown).

**Figure 3:**
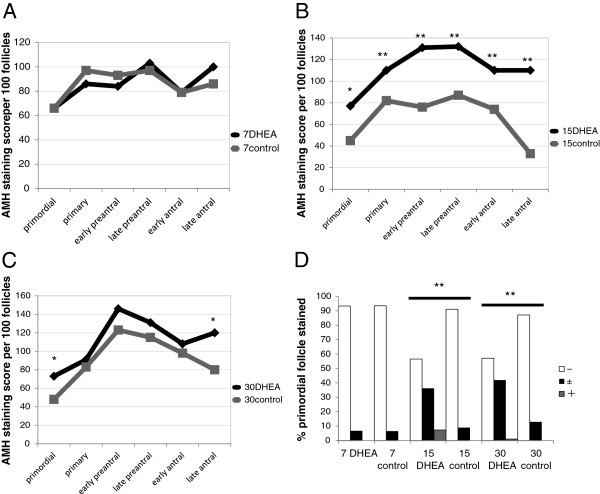
**Comparison of ovarian AMH immunostaining in DHEA-treated and control rats.** AMH was detected immunohistochemically and scored as described in the Methods. **(A)** AMH staining in the day-7 DHEA and control groups. **(B)** AMH staining in the day-15 DHEA and control groups. **(C)** AMH staining in the day-30 DHEA and control groups. **(D)** Percentages of primordial follicles expressing AMH at the indicated levels. Staining intensity was significantly greater than control in the day-15 and day-30 DHEA groups. *P < 0.05; **P < 0.01.

The intensity of AMH staining in the day-7 groups was similar at all follicular developmental stages (Figure 3A). In the day-15 groups, the intensity of staining in the DHEA-treated ovaries was significantly greater in the primordial (P= 0.0079), primary (P = 0.026), early preantral (P = 0.0001), late preantral (P = 0.0014), early antral (p = −0.0048) and late antral (P = 0.0001) follicles (Figure 3B). In the day 30 groups, the intensity of staining in the DHEA group was much greater than control at the primordial (P = 0.0485) and late antral (P = 0.0028) stages.

### Hormone assay

As shown in Table 1, the serum androstendione levels were higher in the DHEA group than the control group on days 15 (P = 0.009) and 30 (P = 0.005). The serum DHEA-S levels were higher in DHEA-treated than controls rats on all days (day 7: P = 0.004, day15: P = 0.009, and day 30: P = 0.009). Serum AMH levels were significantly higher than control in the DHEA group on day 15 (P = 0.04), but were similar in the two groups on days 7 (P = 0.56) and 30 (P = 0.11). AMH values tended to be higher in the DHEA group on day 7, whereas they tended to higher in the control group on day 30.

**Table 1:** Serum hormonal level on days 7, 15 and 30 in DHEA-treated and control rats (Values: median (IQR); ^*^P < 0.05)

	**Day-7 DHEA**	**Day-7 control**	**Day-15 DHEA**	**Day-15 control**	**Day-30 DHEA**	**Day-30 control**
Androstenedione (ng/ml)	0.8 (0.4-0.8)	0.7 (0.5-0.7)	5.6 (3.8-5.6)*	0.2 (0.1-0.5)*	16 (15–21)*	0.3 (0.1-0.3)*
DHEA-s (ng/dl)	4.5 (3.75-5.25)*	< 2*	13 (13–21)*	< 2*	42 (38–72)*	< 2*
AMH (ng/ml)	9.69 (8.79-10.9)	8.48 (7.83-8.98)	5.11 (4.61-6.22)*	3.11 (2.56-3.21)*	1.88 (1.58-2.2)	3.48 (3.26-3.78)

Values: median (IQR); ^*^P < 0.05.

## Discussion

In this study, we found that by day 7, DHEA treatment had caused a slight increase in follicle numbers and serum AMH levels, but it had not affected AMH expression. It is well known that androgens that promote follicle growth most strongly affect preantral to early antral folliculogenesis [17], and it also reportedly stimulates transition from primary to secondary follicles [18]. We speculate that with brief treatment, androgen stimulates follicle development, but does not affect AMH production in granulosa cells. The slight elevation of serum AMH level reflects a slight increase in follicle number.

Medium-term androgen treatment produces the most remarkable changes in the ovaries. Fifteen days of DHEA treatment led to a significant increase in total follicle number and AMH expression in the ovaries, and to significantly greater serum AMH levels. We suggest that the elevation of serum AMH is caused by not only an increase in the number of intermediary follicles, but also by augmented production of AMH in granulosa cells. The increased AMH production is also observed in PCOS [19], which supports the premise that the elevated AMH in PCOS is caused by hyperandrogenemia. Furthermore, the increased AMH changed the folliculogenesis. In early follicle development, AMH reduces primordial and primary follicle growth and prevents depletion of any follicle cohort [10]. We observed that by day15, the increased AMH had inhibited primordial follicle growth, but that follicular development at stages later than primary follicles was maintained by the effect of DHEA. As a result, total follicle number and the proportion of primordial follicles were increased. Although the proportion of intermediary follicles was unchanged, the actual number of intermediary follicles was increased due to the increase in the total follicle number. Consequently, serum AMH should be elevated. Had the elevation in AMH not occurred, follicular development would not have been inhibited, and the follicles would have become depleted [20]. AMH is therefore thought to control the androgen effect on follicular growth.

Long-term treatment caused further morphological changes and led to atresia and atrophy. Thirty days of DHEA treatment caused an increase in the percentages of primordial, primary and atretic follicles, and a decrease in the percentage of intermediary follicles. AMH also reportedly reduces primary follicle growth [21], and we found that growth of primary and primordial follicles was impaired by increased AMH. DHEA would be expected to encourage intermediary follicles to grow to the next stage, but to provide fewer follicles at the intermediary stage. A decrease in the number of intermediary follicles would be expected to reduce serum AMH levels; however, we observed that the reduction in serum AMH was minimal with respect to the decrease in the number of intermediary follicles, because the expressed AMH produced earlier in granulosa cells persisted.

Androgens may regulate follicular atresia [22]; for example, testosterone reportedly increases somatic cell atresia in rat ovaries [23] and antagonizes the anti-apoptotic effects of estradiol in rat granulosa cells in early antral and preantral follicles [24]. However, it was recently reported that androgens also exert an anti-apoptotic effect [25,26]. Thus the relationship between androgens and atresia appears complex. In human studies, increases in the numbers of atretic follicles were observed in the ovaries of androgen-administrated FTM persons [3,8,9]. The treatment periods were fairly long, averaging 38 months [3], 21 months [8] and 35 months [9], respectively. Consistent with those reports, we observed increases in the numbers of atretic follicles with long-term DHEA treatment. Because long-term administration of DHEA likely results in follicular atresia, administration of DHEA to patients, which is aimed at increasing follicles in cases of primary ovarian insufficiency [27], may have the potential to cause atresia when administered for long periods.

In our study, we observed weak expression of AMH in primordial follicles. This is in contrast to two earlier reports, where no AMH expression was observed in primordial follicles [28,29]. Consistent with our finding, immunohistochemical staining by Stubbs et al. revealed expression of AMH in primordial follicles [13]. They also reported that significantly fewer primordial follicles stained positively for AMH in PCOS ovaries than non-PCO ovaries. They suggested that reduced local exposure to AMH would result in a higher proportion of primordial follicles initiating growth in PCOS. Primordial and early growing follicles lack a direct blood supply [7], and local paracrine signals play an important part in regulating initiation of follicle growth [13]. In our study, administration of androgens increased production of AMH, which regulates the growth of primordial follicles. Consequently, androgen-induced AMH expression provides local negative feedback affecting folliculogenesis augmented by androgen. The DHEA-treated PCOS model differs from true PCOS in that regard. The pathogenesis of PCOS is thought to involve an increase in the initiation, subsequent growth and arrest of follicle maturation [7]. However, the pathogenesis in the DHEA-induced PCOS model promotes follicular growth and atresia regulated by enhanced AMH. Only with medium-term DHEA administration (increasing follicle phase) is ovarian morphology in the rat PCOS model similar to that of true PCOS.

## Conclusions

We conclude that medium-term androgen administration increases the number of follicles in rat ovaries and enhances production of AMH. However, increased AMH expression affects early follicles by interfering with their development. Consequently, long-term androgen administration leads to a decrease in healthy follicles and an increase in atretic follicles, which contributes to ovarian atrophy.

## Abbreviations

PCOS: Polycystic ovarian syndrome; AMH: Anti-Mullerian hormone; DHEA: Dehydroepiandrosterone; FTM: Female-to-male transsexual.

## Competing interests

The authors declare that they have no competing interests.

## Authors’ contributions

KI participated in the design and performance of the experiments, analysis and interpretation of the data, and drafting the manuscript. TB conceived the project, designed experiments and revised the manuscript. MM was involved in data analysis. HH supervised the animal studies and contributed to the preparation of the manuscript. TE was involved in the conception and design of the study and reviewed the manuscript. TK was involved in the conception and design of the study. TS was involved in the conception of the study, reviewed the manuscript, and approved the final draft. All authors read and approved the final manuscript.
